# Stereotactic Body Radiotherapy of Colorectal Cancer Oligometastases to the Liver: Three Years Follow-Up

**DOI:** 10.3390/cancers17172823

**Published:** 2025-08-28

**Authors:** Alexey Moskalenko, Marina Chernykh, Damir Ichshanov, Ksenia Malinina, Anna Ikonnikova, Vladimir Lyadov

**Affiliations:** 1 Moscow State Budgetary Healthcare Institution “Moscow City Hospital Named After S.S. Yudin, Moscow Healthcare Department”, 117152 Moscow, Russia; vlyadov@gmail.com; 2 Department of Radiotherapy, N.N. Blokhin National Medical Research Center of Oncology of the Ministry of Health of the Russian Federation, 115478 Moscow, Russia; dr.chernich@mail.ru (M.C.); dgi@kantoku.ru (D.I.); 3 Limited Liability Company “PET-Technology Podolsk”, 142110 Podolsk, Russia; k.malinina@oncoart.ru; 4 Laboratory of Biological Microchips, Engelhardt Institute of Molecular Biology, Russian Academy of Sciences, 119991 Moscow, Russia; anyuik@gmail.com; 5 Department of Oncology and Palliative Medicine Named After Academician A.I. Savitsky, Russian Medical Academy of Continuous Professional Education, 123242 Moscow, Russia; 6 Department of Oncology, Novokuznetsk State Institute for Postgraduate Medical Education, 654005 Novokuznetsk, Russia

**Keywords:** SBRT, stereotactic body radiation therapy, colorectal cancer, oligometastases, radiotherapy

## Abstract

Background: Liver resection remains the gold standard treatment for colorectal cancer (CRC) liver metastases, while stereotactic body radiotherapy (SBRT) offers an alternative for patients with unresectable metastases. However, the precise indications for SBRT, optimal radiation doses, and treatment regimens have yet to be definitively established. Methods: A total of 91 patients with 152 lesions underwent SBRT, receiving a total dose ranging from 40 to 60 Gy delivered in 4–5 fractions per lesion, with a median dose of 50 Gy. Results: The three-year local control (LC) and overall survival (OS) rates were 62.6% and 45.1%, respectively. No cases of Grade ≥ 3 toxicity were observed. Factors negatively affecting LC included metastasis diameter ≥ 2.7 cm and number of metastases ≥ 3, with hazard ratios (HR) of 2.73 and 2.24, respectively. A biologically effective dose (BED) of ≥137.7 Gy was associated with a significant improvement in local control (LC) (HR 0.25), a finding that was also confirmed by the inverse probability of treatment weighting (IPTW) analysis. Significant predictors for poorer OS included RAS gene mutations, metastasis diameter ≥ 2.6 cm, and synchronous metastases, with HRs of 2.27, 2.03, and 2.11, respectively. Landmark analysis demonstrated that local recurrence within 12 months after SBRT significantly reduced OS (HR 2.68). Conclusions: SBRT is a safe and effective method for achieving local control of CRC liver oligometastases. Further research is warranted to optimize treatment protocols and refine patient selection criteria.

## Simple Summary

Many patients with colorectal liver metastases are not candidates for surgical treatment due to the unresectable nature of their tumors. Stereotactic body radiation therapy (SBRT) is a promising alternative for local tumor control; however, experience with this method in such patients remains limited. We present the results of a three-year follow-up study involving 91 patients. Our findings show that higher radiation doses lead to better local control (LC), and that LC rates were higher in patients with fewer and smaller metastases. Furthermore, landmark analysis revealed that local recurrence within the first year after SBRT significantly reduced overall survival. Overall survival was also poorer in patients with *RAS* mutations, synchronous metastases, and a greater number and larger size of metastases.

## 1. Introduction

Colorectal cancer (CRC) ranks third in the global landscape of cancer incidence, accounting for 9.8% of cases, and second in mortality, representing 9.2% of cancer-related deaths. In 2022, there were over 1.85 million new cases diagnosed and more than 850,000 deaths attributed to CRC [1]. Among newly diagnosed patients, 20% to 50% present with synchronous metastases [2,3], with the liver and lungs being the most common sites for metastasis [4,5]. In as early as 1995 Hellman and Weichselbaum suggested to define limited metastatic process as oligometastases characterizing it as the presence of 1 to 5 metastases in one or more organs [6]. Patients with oligometastatic disease may benefit from a multidisciplinary treatment approach that combines local control (LC) of metastases with systemic chemotherapy to enhance overall survival (OS) [7].

Combined treatment modalities, such as liver resection accompanied by systemic chemotherapy, have demonstrated the potential to achieve a 5-year OS rate exceeding 40% in patients with oligometastatic disease [8]. However, liver resection is feasible in only 10% to 25% of patients at the time of diagnosis because of the location of lesions, poor functional status of the liver parenchyma and/or patient’s somatic status [5,7]. For those patients who are medically inoperable or decline surgery, various types of local therapies, such radiofrequency ablation, microwave ablation and other similar modalities were developed. Nowadays extracranial stereotactic body radiotherapy (SBRT) is considered a valid alternative to ablation due to its non–invasive nature, good tolerance among patients and high local control rates [3,4,9].

Despite the number of studies devoted to SBRT for CRC oligometastases in the liver increases, the indications for its use, radiation doses and treatment regimens are not yet decisively determined. For instance, some studies include patients with both liver and lung metastases or other primary tumor sites [10,11,12]. Dose of radiation is another source of heterogeneity: Scorcetti et al. [13] carried out SBRT in 75 Gy in 3 fractions, while Voglhuber et al. [14] applied the mode of 35 Gy in 5 fractions. Petrelli et al. [9] managed to show in their meta-analysis including 18 studies and 656 patients that a biologically effective dose (BED) exceeding 100 Gy is associated with better LC. Unfortunately, various studies include patients with different number and size of metastases which makes the efficacy assessment difficult [15,16]. Thus, while SBRT represents a significant advancement in the management of unresectable liver metastases, efforts are necessary to define optimal irradiation strategies and identify patients who benefit most from this method of therapy.

The objectives of our study are to evaluate immediate and long-term outcomes of SBRT and to identify predictors of its efficacy in patients with oligometastases of CRC in the liver.

## 2. Materials and Methods

### 2.1. Patients and Study Design

This retrospective study included 91 patients with oligometastases of CRC in the liver who underwent SBRT at the N.N. Blokhin National Medical Research Center of Oncology (*n* = 50) and “PET-Technology Podolsk” clinic (*n* = 41) from 2018 to 2022. All patients received chemotherapy at the N.N. Blokhin National Medical Research Center of Oncology. The inclusion criteria were as follows: presence of 1 to 5 liver metastases from CRC, absence of uncontrolled extrahepatic metastases, Eastern Cooperative Oncology Group (ECOG) performance status of 0 to 2, age ≥ 18 years, and provision of written informed consent. Previous or concomitant systemic therapies were permitted. All patients underwent imaging evaluation using triple-phase computed tomography (CT) or positron emission tomography (PET) scans.

Each case was reviewed by a multidisciplinary tumor board, including a dedicated liver surgeon. Patients were deemed unsuitable for hepatic resection due to one or more of the following reasons: small liver remnant after prior liver resection (*n* = 33), lesion location precluding safe surgery (*n* = 36), or significant comorbidities (*n* = 22). All patients had undergone primary tumor resection prior to SBRT treatment of hepatic metastases.

The study was conducted in accordance with the Declaration of Helsinki and was approved by the Local Ethics Committee of Moscow City Hospital named after S.S. Yudin (protocol No. 15–2022, approved on 2 February 2022).

### 2.2. Methodology of SBRT

Liver metastasis within the boundaries determined by imaging methods was taken as the “gross tumor volume” (GTV) which also corresponded to “clinical target volume” (CTV) for highly and moderately differentiated CRC. In patients with poorly differentiated tumors the CTV margin was increased by 5–10 mm within the liver to include the zone of potential tumor cell infiltration around the metastasis in the irradiation volume. The “planning target volume” (PTV) was automatically generated by determining the margins from the CTV. The craniocaudal margins were 10 mm and the radial margins were 5 mm.

SBRT was performed under image–guided radiotherapy (IGRT) on a Varian Clinac 2300 iX electron accelerator (Varian Medical Systems, Palo Alto, CA, USA) using three–dimensional planning (3 DCRT), a Millennium 120 multileaf collimator and dynamic wedge filters with 6 MeV photons. RPM (Real–time Position Management) to exclude the influence of respiratory movements by holding the breath during inspiration. GE Light Speed spiral CT (GE LightSpeed VCT GE Healthcare Technologies, Inc., Chicago, IL, USA) was used for topometry with subsequent magnetic resonance imaging using Fusion technology to select the volume of irradiation. Verification of the external beam radiation therapy plan was carried out using cone–beam computed tomography technology (Cone–Beam CT) and x–ray images in a kilovoltage beam on the Clinac 2300 iX accelerator. The patient was positioned using an individual fixation device. Laser Guard patient protection technology was used during external beam radiation therapy sessions.

All the patients were treated according to respective institutional guidelines. The biologically effective dose (BED) was calculated using the linear quadratic model as BED = D × (1 + d/α/β) where D is the total dose, d is the dose per fraction and the α/β ratio for the tumor was 10 Gy. BED was used to compare the outcomes of various treatment protocols with different fraction sizes and doses.

SBRT was performed in a hypofractionation mode, achieving a total dose of 40 to 60 Gy per lesion in 3–5 fractions with a median of 50 Gy in 5 fractions that was equal to biologically effective dose (BED) of 100 Gy. 46 patients received BED ≤ 100 Gy (80–100 Gy) and 45 patients had BED ≥ 137.7 Gy (137.7–180 Gy).

All the existing metastases were irradiated during one treatment session. In the absence of progression, patients continued dynamic observation; in case of progression, chemotherapy was administered; some patients with oligoprogression were re-considered as candidates for local treatment.

### 2.3. Patient Characteristics

A total of 91 patients with 158 metastases were included in the study (Table 1). Among them, 45 patients (49.5%) had solitary liver metastases, 30 patients (33%) had two metastases, 14 patients (15.4%) had three metastases, and two patients each had four and five metastases, respectively. Extrahepatic CRC metastases were previously treated in 24 (26.4%) patients: 17 (18.7%) had single lesions in the lungs that had been surgically removed previously; 5 (5.5%)—metastases in solitary retroperitoneal lymph node, treated with SBRT; 1 (1.1%)—solitary metastasis to the body of the 5th lumbar vertebra (SBRT was also performed) and one patient 1 (1.1%) had a solitary metastasis into the spleen, that had been surgically removed. Mutations in genes of the RAS pathway were present in 30% of patients. The majority of patients (*n* = 88) received at least one line of systemic chemotherapy, with 63 patients (69.2%) receiving two or more lines of chemotherapy. Prior to SBRT, liver resection had been performed in 67 patients (73.6%): 49 patients (73.1%) underwent one liver resection, 14 patients (20.9%) underwent two resections, 3 patients (4.5%) underwent three resections, and 1 patient (1.5%) underwent four consecutive atypical liver resections. The time to onset of metachronous metastases ranged from 10 months to 4.5 years with a median of 1.5 years. For classification of oligometastatic disease, we adapted the system proposed by Guckenberger et al. [17], simplifying it into three strata: de novo, induced, and recurrent metastases.

### 2.4. Endpoints and Assessment Methods

Study endpoints were post-radiation toxicity and long-term results, including LC and OS. After the local treatment of metastases patients underwent dynamic monitoring: CT or MRI of the abdominal organs with intravenous bolus contrast every 3 months. LC was assessed according to EORTC-RECIST 1.1 criteria [18]. LC was defined as the status of the lesion treated with SBRT. The development of liver metastases outside this treatment zone was not considered an LC failure. Toxicity was assessed according to Common Terminology Criteria for Adverse Events (CTCAE) 5.0 [19].

Statistical analysis and visualization were performed using R version 4.3.3 (R Foundation for Statistical Computing, Vienna, Austria), using the packages “survival”, “survminer”, “twang”, “ggsurvfit” and “ggplot2”. Statistical significance was defined as a *p*-value of less than 0.05. Categorical variables were compared using two-sided Fisher’s exact test and continuous variables with the Mann–Whitney U test. The Kaplan–Meier method was used to construct survival curves for LC and OS, comparison between groups was carried out by log-rank test. The influence of studied factors on long-term treatment outcomes was assessed using the Cox proportional hazards model in a univariate analysis, and predictors with a *p*-value < 0.05 were subsequently included in a multivariate analysis. Two patients with unknown *RAS* status were excluded from the univariate analysis of LC and OS and the multivariate analysis of OS. To facilitate the visualization and interpretation of the impact of quantitative variables on long-term treatment outcomes, we categorized the groups based on threshold values determined using the “surv_cutpoint” function from the “survminer” package. The inverse probability of treatment weighting (IPTW) approach was applied to analyze the effect of dose (differences between groups with BED ≤ 100 Gy and ≥137.7 Gy) on LC, to correct for the imbalance between groups. We considered a standardized effect size not exceeding 0.1 as the criterion for sufficient balance of covariates between groups. Furthermore, we tested the impact of LC on OS in a landmark analysis among patients who were alive at 12 months, comparing patients with and without local recurrence during this period.

## 3. Results

### 3.1. Toxicity

SBRT sessions were performed on an outpatient basis, daily, and were generally well tolerated. All patients completed the full course of radiation therapy. Grade 1–2 toxicity, manifested in the form of nausea, low-grade fever, pain, diarrhea, and general malaise, was observed in 22 (24.2%) patients. The conditions mentioned above were self-limiting in most cases. No instances of grade 3 or higher toxicity, including cases of radiation-induced hepatotoxicity, were observed.

### 3.2. Long-Term Results

Median follow-up was 32.2 months. In the entire study group the 1-year LC was 67.7% (95% CI 57.8–79.4%), 2- and 3-year rates were identical and were 53.8% (95% CI 42.7–67.6%) with a median of 50.7 months. The 1-year overall survival was 94.0% (95% CI 89.0–99.2), 2-year survival was 66.7% (95% CI 57.8–79.4%), and 3-year survival was 45.1% (95% CI 34.5–59.1), with a median of 30.7 months. We further analyzed LC and OS based on potentially important predictors, including sex, age, number of previous chemotherapy lines, ECOG-status, mutations in *RAS* genes, diameter and number of metastases, and radiation dose.

#### 3.2.1. Local Control

Radiation dose, number, and diameter of metastases were significant predictors for LC in the univariate analysis (Table 2). BED ≥ 137.7 Gy was a favorable factor for LC, whereas an increased number and diameter of metastases were associated with worse LC. According to the defined thresholds for quantitative variables (the number of metastases and maximum metastasis diameter), the following subgroups were identified: 1–2 metastases versus 3 or more, and a diameter greater than 2.7 cm versus less than or equal to 2.7 cm. Those variables were further used in the multivariate analysis along with the radiation dose BED (≥137.7 Gy vs. ≤100 Gy). In the multivariate analysis, all these predictors were also significant, indicating their independent impact on the LC rate (Table 2).

We then applied the IPTW approach, including the BED ≤ 100 Gy and ≥137.7 Gy as a treatment option and the diameter and number of metastases as covariates. Initially, there was a moderate imbalance between the groups, which was corrected by applying IPTW (Table S1). Cox regression analysis of the weighted data revealed that radiation dose, diameter and number of metastases significantly impacted the LC rate: BED ≥ 137.7 Gy significantly predicted better LC (HR 0.25, 95% CI: 0.12–0.55) while the number of metastases > 2 and the size of metastases > 2.7 cm predicted worse LC (HR 2.24, 95% CI 1.05–4.77 and HR 2.73, 95% CI 1.32–5.59, respectively) (Table S2).

**Figure 1:**
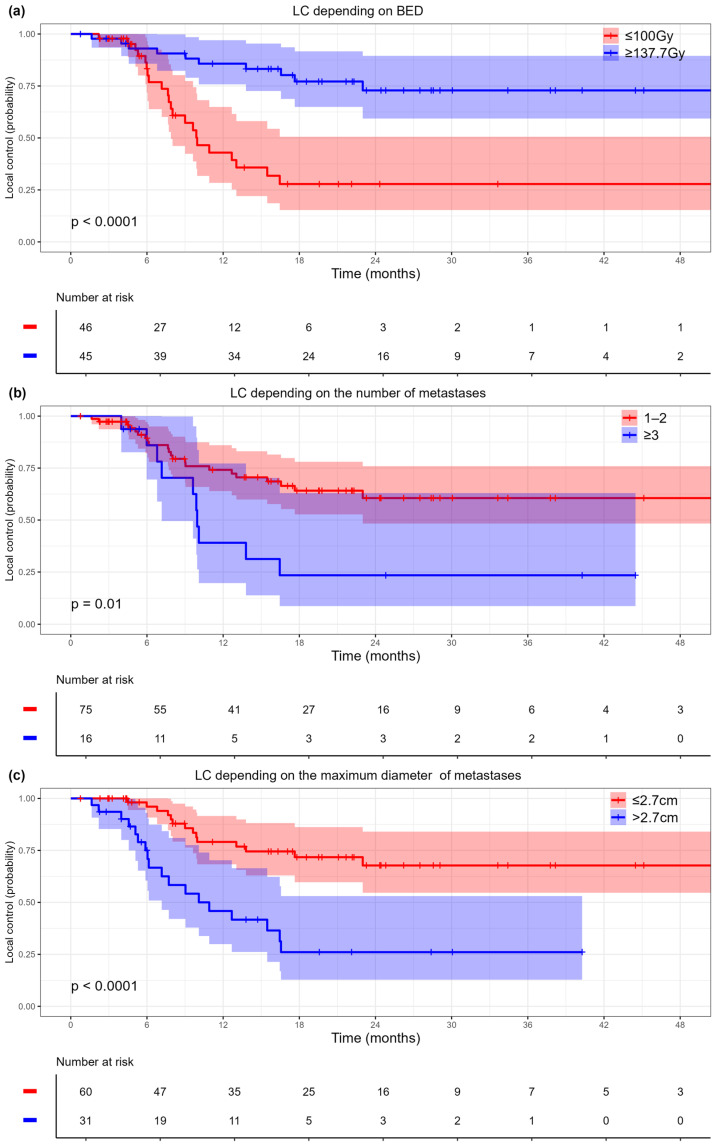
Kaplan–Meier curves for local control: (**a**) by radiation dose, (**b**) by diameter of metastases, (**c**) by number of metastases. BED: biologically effective dose. Shaded areas represent 95% confidence intervals. Corresponding risk tables display patient counts and event numbers at selected time points.

The Kaplan–Meier curves for LC based on the dose, the size and the number of metastases are shown in Figure 1. The one-, two-, and three-year LC rates, along with the medians for each group, are presented in Table S3. In the group of patients with BED ≥ 137.7 Gy, the three-year LC rate was 72.9% (95% CI = 59.3–89.5%). In contrast, for the group with a BED of ≤100 Gy, the three-year LC rate was 27.8% (95% CI = 13.3–50.6%), with the median achieved only in the latter group at 9.95 months (Figure 1a). The three-year LC rate for metastases smaller than 2.7 cm was 67.7% (95% CI = 54.6–84%), while for metastases measuring ≥ 2.7 cm, it was 26% (95% CI = 12.8–53%) (Figure 1b). The LC rate for patients with 1–2 metastases was 62.9% (95% CI = 48.5–81.6%), compared to 45.1% (95% CI = 30.3–67.3%) for patients with ≥3 metastases (Figure 1c).

It is noteworthy that progression patterns differed significantly between dose groups: while the BED ≥ 137.7 Gy cohort primarily developed extra-target lesions (10/42 with local failure), the BED ≤ 100 Gy group had a 57.5% local failure rate (23/40).

#### 3.2.2. Overall Survival

In the univariate analysis, significant risk factors for OS were mutations in the *RAS* genes, the presence of synchronous metastases as well as the diameter and the number of metastases. Next, we determined the cutoff points and identified groups for the quantitative predictors: for the number of metastases, the value was 1–2 versus 3 or more, while for the size of the metastases the threshold was set at 2.6 cm. In multivariate analysis, the following predictors remained significant: mutations in the *RAS* system genes, synchronous metastases and the size of the metastasis > 2.6 cm (Table 3).

**Figure 2:**
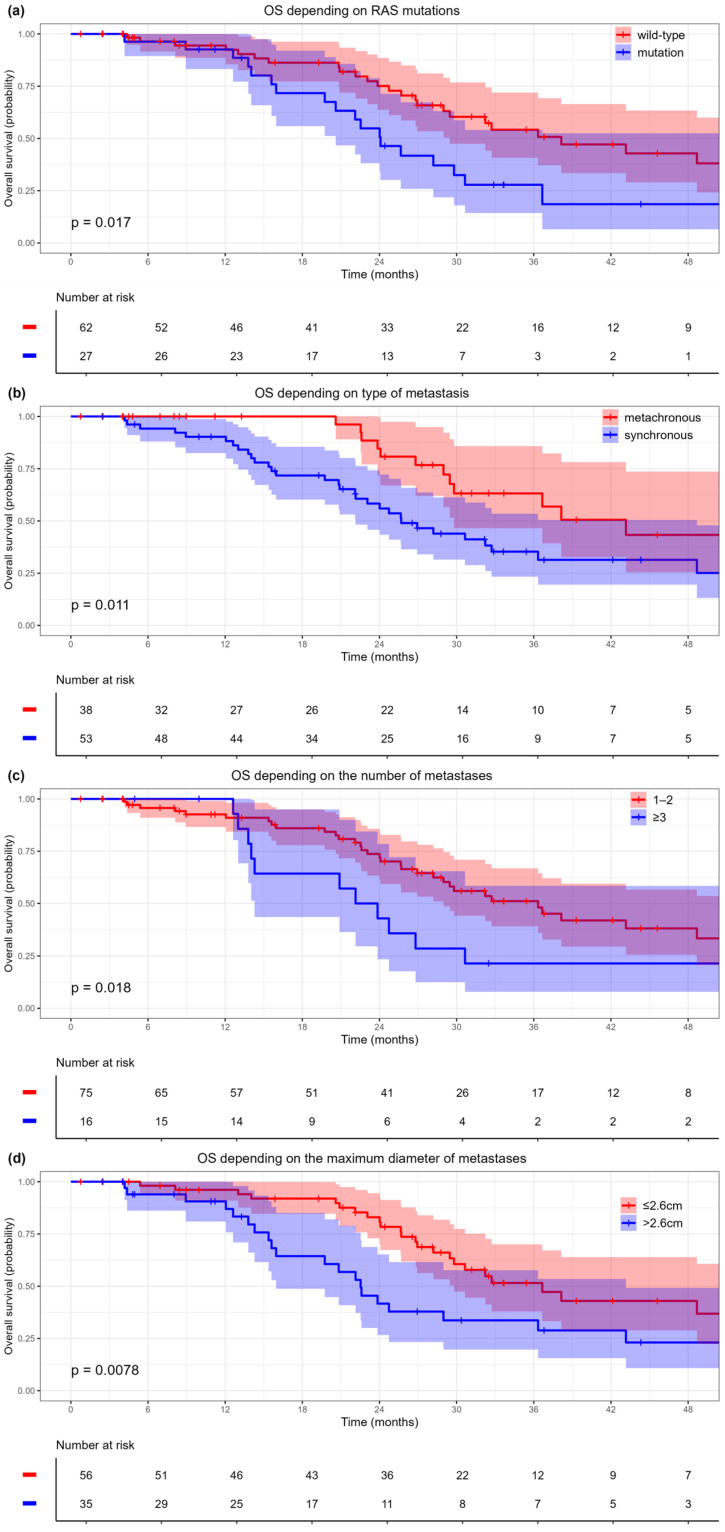
Kaplan–Meier curves for overall survival: (**a**) by *RAS* mutation status, (**b**) by metastasis type, (**c**) by number of metastases, (**d**) by metastasis size. Shaded areas represent 95% confidence intervals. Corresponding risk tables display patient counts and event numbers at selected time points.

The Kaplan–Meier curves for OS based on *RAS* status, the type, the size and the number of metastases are shown in Figure 2. The one-, two-, and three-year OS rates, along with the medians for each group, are presented in Table S4.

**Figure 3:**
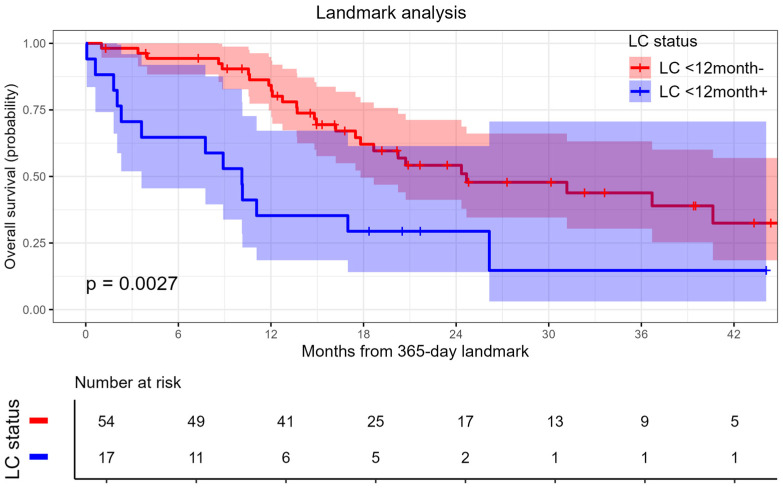
Landmark analysis: impact of local recurrence on overall survival. Corresponding risk table displays patient counts and event numbers at selected time points.

A landmark analysis was performed as described by Stera et al. [20]. Among patients who were alive at 12 months after SBRT, local recurrence occurring during this time was shown to be significantly associated with worse OS (Figure 3), HR 2.68 (95% CI 1.37–5.26, *p* = 0.003).

## 4. Discussion

SBRT is increasingly recognized as an efficient method of local control in patients with liver metastases [9]. However, there remains a lack of clarity regarding how to identify patients who would benefit most from this approach, and the optimal SBRT regimen has yet to be established. The main goal of SBRT is to achieve a high rate of tumor LC, which might further translate into improved OS. The primary objective of this study was to identify predictors of LC and OS in patients with CRC liver metastases treated with SBRT, focusing on the characteristics of metastatic lesions and the radiation dose delivered.

An important advantage of SBRT is its low incidence of severe toxicity. In many studies, there is a reported absence of grade 3 or higher toxicity [12,21], with occurrences where such toxicity was noted being around 5% [9,16,22,23]. In our study, we confirmed the high tolerability of this method, as no cases of toxicity of grade 3 or higher were observed.

The good tolerability of SBRT allows for dose escalation, which can lead to improved treatment outcomes. This is particularly significant for CRC metastases in the liver. Several studies have explored the application of SBRT in patients with liver metastases from various primary tumors [20,21] and, on the other hand, in patients with CRC metastases to various organs [10,24,25], primarily the liver and lungs. In this context, both colorectal histology [20] and the localization of metastases in the liver [24] (as compared to the lungs) are associated with radioresistance and linked to worse LC rates.

Several studies consistently indicate that dose escalation is associated with improved local control (LC) [25,26,27,28], which was also demonstrated in our study: the three-year LC rate was 72.9% in patients with a BED of ≥137.7 Gy and 27.8% in those with a BED of ≤100 Gy. A meta-analysis by Petrelli et al. [9] showed a correlation between BED > 100 Gy and better LC rates (*p* = 0.001; R = 0.47). Scorsetti [13] managed to use SBRT in a selected group of patients in a 75 Gy regimen in 3 fractions (BED = 262.5 Gy) with a 2-year LC of 91% in the absence of grade 3 toxicity. Therefore, radiation dose escalation seems promising.

Regarding the impact of radiation dose on OS, the data is somewhat ambiguous. In our study, OS did not show a statistically significant difference based on the BED. However, there is evidence suggesting that a higher radiation dose has a positive effect on OS. Meta-analysis of Petrelli [9] et al. demonstrated a weak but statistically significant correlation between BED > 100 Gy and OS (*p* = 0.001; R = 0.29). Some other studies have also shown that exceeding BED > 100 Gy is associated with better OS [25,27]. Further analysis of the effect of radiation dose on OS needs methodologically sound studies with homogeneous groups of patients regarding the size and the number of metastases and the amount of systemic therapy.

Another important predictor of LC and OS in our study was the size of the metastasis: larger metastases were associated with a worse prognosis. It should be mentioned that in various studies, both the diameter and the volume of the tumor, which in turn corresponds to the GTV, are used as a characteristic of the size of the metastatic focus. In this study, we used the diameter of the metastasis as the simplest indicator. The dependence of the LC rate on the size of the metastatic lesion has been shown by several authors [14,27]. The threshold values for the diameter of metastasis determined in our study were 2.7 cm for LC and 2.6 cm for OS. These values are quite similar to those reported in other studies, which identified the size of metastases ranging from 2.45 cm to 3 cm to be cutoff values for LC failure [28,29]. Studies using GTV as a predictive marker of LC also showed that larger volume of metastases was associated with worse LC and/or OS [26,27,30].

The number of metastases as a predictor of SBRT efficacy has primarily been studied in relation to OS, whereas it is less frequently included in LC analyses. Despite several authors demonstrating a lack of association between the number of metastatic lesions and LC [31] or OS [20,24,31], our data suggest that the number of metastases may serve as a risk factor for both LC and OS, with three metastases being the cutoff. We believe that one possible reason for this association might be the complexity of planning radiation therapy in patients with multiple liver nodules while adhering to dose and volume constraints for the liver and surrounding organs at risk. Further research may clarify the prognostic role of the number of metastases.

The deterioration of OS in the presence of synchronous metastases [32] and mutations in the genes of the *RAS* system has been studied quite well, we have also used these predictors as covariates in the multivariate analysis of OS.

Some data suggest the possibility of increasing OS due to the high level of LC demonstrated by SBRT. McPartlin et al. [16] also showed worsening OS in patients with local progression of metastases after SBRT (HR 2.16; *p* = 0.001). Our data indicate that patients who experienced local recurrence within the first 12 months following SBRT exhibited significantly worse OS with a HR 2.68 (95% CI 1.37–5.26, *p* = 0.003). These findings are consistent with prior studies that have demonstrated poorer OS in patients with rapid local recurrence after SBRT [15,20,30].

Based on our results and previous studies, we believe that SBRT will achieve the best results in patients with 1–2 metachronous CRC metastases of 3 cm or less in diameter and without RAS mutation.

Our study has some evident limitations: retrospective design, multicenter patient recruitment, and heterogeneous patient population. It is important to say that SBRT as a treatment modality is usually considered when other therapeutic options have been exhausted, which worsens the survival rate. Despite there are several large patient series and meta-analyses no commonly accepted indications exist today regarding the best indications for SBRT in CRC with liver metastases. We feel that applying more strict inclusion criteria based on the predictive biomarkers to further prospective trials would probably make the progress in the field more substantial.

## 5. Conclusions

SBRT is a safe and efficient method of local control in patients with oligometastatic colorectal cancer with liver metastases.

Further prospective research is required to individualize the indications for this treatment method and optimize its methodology.

## Figures and Tables

**Table 1:** Patient characteristics.

Characteristics	All Patients (*n* = 91)	BED ≤ 100 Gy (*n* = 46)	BED ≥ 137.7 Gy (*n* = 45)	*p*–Value
Sex (female), *n* (%)	45 (49.5%)	22 (47.8%)	23 (51.1%)	0.834
Age (years), median (min–max)	62 (27–85)	62 (31–85)	61 (27–83)	0.249
ECOG, *n* (%):				
0–1	65 (71.4%)	30 (65.2%)	35 (77.8%)	0.246
2	26 (28.6%)	16 (34.8%)	10 (22.2%)	
pT, *n* (%):				
2	5 (5.5%)	1 (2.2%)	4 (8.9%)	0.477
3	53 (58.2%)	28 (60.9%)	25 (55.6%)	
4	33 (36.3%)	17 (37%)	16 (35.6%)	
pN, *n* (%):				
0	27 (29.7%)	13 (28.3%)	14 (31.1%)	0.830
1	41 (45.1%)	20 (43.5%)	21 (46.7%)	
2	23 (25.3%)	13 (28.3%)	10 (22.2%)	
*RAS* mutation ^1^, *n* (%)	62 (69.7%)	32 (72.7%)	30 (66.7%)	0.645
Synchronous metastases, *n* (%)	38 (41.8)	21 (45.7)	17 (37.8)	0.525
Number of metastases, *n* (%):				
1	45 (49.5%)	20 (43.5%)	25 (55.6%)	0.524
2	30 (33%)	17 (37%)	13 (28.9%)	
≥3	16 (17.6%)	9 (19.6%)	7 (15.6%)	
Diameter of metastasis (cm), median (min–max)	2.5 (0.8–6.5)	2.5 (0.8–7)	1.9 (0.8–6.5)	0.075
Previous liver resection, *n* (%)	67 (73.6)	32 (69.6)	35 (77.8)	0.477
Prior chemotherapy (lines), median (min–max)	2 (0–4)	2 (0–3)	2 (0–4)	0.813
Extrahepatic metastases, *n* (%)	24 (26.4)	11 (23.9)	13 (28.9)	0.639
Type of oligometastases ^2^, *n* (%):				
De novo	24 (26.7%)	14 (31.1%)	10 (22.2%)	0.467
Induced	20 (22.2%)	11 (24.4%)	9 (20%)	
Repeated	46 (51.1%)	20 (44.4%)	26 (57.8%)	

^1^ RAS status was not available for 2 patients. ^2^ Classification of oligometastases by Gukenberger [17]. ECOG: Eastern Cooperative Oncology Group performance status.

**Table 2:** Univariate and multivariate analyses for local control.

	Univariate Analysis		Multivariate Analysis *	
Predictor	HR (95% CI)	*p*-Value	HR (95% CI)	*p*-Value
Sex (male vs. female)	1.78 (0.89–3.57)	0.104		
Age, years	1 (0.97–1.02)	0.799		
Prior chemotherapy, lines	1.17 (0.83–1.67)	0.37		
ECOG (2 vs. 0–1)	1.02 (0.46–2.28)	0.961		
*RAS* mutation	1.82 (0.9–3.69)	0.096		
Microsatellite instability	0.62 (0.08–4.51)	0.633		
pT	1.23 (0.68–2.25)	0.495		
pN (1–2 vs. 0)	1.18 (0.55–2.53)	0.68		
Extrahepatic metastases	0.69 (0.3–1.59)	0.387		
Previous liver resection	1 (0.45–2.22)	0.999		
Type (synchronous vs. metachronous)	1.18 (0.58–2.42)	0.642		
Diameter of metastasis, cm	1.38 (1.13–1.68)	0.001		
Diameter of metastasis (>2.7 cm vs. ≤2.7 cm)	3.77 (1.86–7.63)	<0.001	2.73 (1.32–5.59)	<0.001
The number of metastases	1.51 (1.07–2.12)	0.018		
The number of metastases (≥3 vs. 1–2)	2.59 (1.22–5.49)	0.013	2.24 (1.05–4.77)	0.037
BED (≥137.7 Gy vs. ≤100 Gy)	0.21 (0.1–0.45)	<0.001	0.25 (0.12–0.55)	<0.001

* Concordance = 0.764 (se = 0.037), logrank test = 33.95 on 3 df, *p* < 0.001.

**Table 3:** Univariate and multivariate analyses for overall survival.

	Univariate Analysis		Multivariate Analysis *	
Predictor	HR (95% CI)	*p*–Value	HR (95% CI)	*p*–Value
Sex (male vs. female)	1.03 (0.56–1.87)	0.93		
Age, years	1.01 (0.99–1.03)	0.33		
Prior chemotherapy, lines	1.2 (0.89–1.61)	0.23		
ECOG (2 vs. 0–1)	1.56 (0.79–3.07)	0.2		
*RAS* mutation	2.09 (1.12–3.88)	0.02	2.27 (1.21–4.25)	0.01
Microsatellite instability	1.04 (0.25–4.34)	0.953		
pT	1.04 (0.62–1.74)	0.887		
pN (1–2 vs. 0)	0.85 (0.46–1.57)	0.597		
Extrahepatic metastases	1.07 (0.55–2.08)	0.843		
Previous liver resection	1.79 (0.86–3.73)	0.12		
Type (synchronous vs. metachronous)	2.31 (1.19–4.49)	0.013	2.11 (1.04–4.26)	0.037
Diameter of metastasis, cm	1.25 (1.03–1.52)	0.026		
Diameter of metastasis (>2.6 cm vs. ≤2.6 cm)	2.2 (1.21–3.98)	0.009	2.03 (1.07–3.83)	0.03
The number of metastases	1.36 (1.03–1.81)	0.032		
The number of metastases (≥3 vs. 1–2)	2.2 (1.12–4.26)	0.022	1.85 (0.93–3.63)	0.076
BED (≥137.7 Gy vs. ≤100 Gy)	1.13 (0.61–2.1)	0.688		

* Concordance = 0.726 (se = 0.04), logrank test = 22.37 on 4 df, *p* < 0.001.

## Data Availability

The data presented in this study are available from the corresponding author upon reasonable request.

## Supplementary Materials

The following supporting information can be downloaded at: https://www.mdpi.com/article/10.3390/cancers17172823/s1, Table S1: Balance characteristics between groups before and after inverse probability of treatment weighting; Table S2: Cox regression results after inverse probability of treatment weighting adjustment; Table S3: Local control depending on risk factors; Table S4: Overall survival depending on risk factors.
