# Supplementary material for: Stereotactic Body Radiotherapy of Colorectal Cancer Oligometastases to the Liver: Three Years Follow-Up
Source: Cancers (Basel). 2025 Aug 28;17(17):2823. doi: 10.3390/cancers17172823 (PMC12427416; doi:10.3390/cancers17172823)
Supplement: Supplementary file 1 [file cancers-17-02823-s001.zip › cancers-3827898-supplementary.pdf]

Table S1. Balance Characteristics Between Groups Before and After Weighting

		BED ≤100 Gy, mean (sd)	BED ≥137.7 Gy, mean (sd)	Standardized Effect Size	p-value
**Unweighted**					
Number of metastases	1-2	0.844 (0.362)	0.804 (0.397)	0.105	0.619
	≥ 3	0.156 (0.362)	0.196 (0.397)	-0.105	
Diameter of metastasis	≤2.7cm	0.733 (0.442)	0.587 (0.492)	0.309	0.146
	>2.7cm	0.267 (0.442)	0.413 (0.492)	-0.309	
**After Weighting**					
Number of metastases	1-2	0.82 (0.384)	0.826 (0.379)	-0.015	0.945
	≥3	0.18 (0.384)	0.174 (0.379)	0.015	
Diameter of metastasis	≤2.7cm	0.656 (0.475)	0.662 (0.473)	-0.013	0.95
	>2.7cm	0.344 (0.475)	0.338 (0.473)	0.013	

BED – biological effective dose.

Table S2. Cox Regression Results After IPTW Adjustment

Predictor	HR (95% CI)	p-value
BED ≥ 137.7 Gy	0.28 (0.13 - 0.62)	0.00168
Number of metastases (≥ 3 vs 1–2)	2.21 (1.02 - 4.79)	0.04518
Diameter of metastasis ≥2.7cm	2.81 (1.39 - 5.69)	0.0042
Concordance= 0.738 (se = 0.041), Score (logrank) test = 56.6 on 3 df, p <0.001		

Table S3. Local control depending on risk factors

Predictor		1 year	2 year	3 year	Median
BED	≤100 Gy	42.9 (28.4 – 64.9)	27.8 (15.3 – 50.6)	27.8 (15.3 – 50.6)	9.95
	≥137.7 Gy	85.7 (75.8 – 97)	72.9 (59.3 – 89.5)	72.9 (59.3 – 89.5)	NA
Number of metastases, *n*:	1–2	74.1 (63.9 – 86)	60.6 (48.3 – 75.9)	60.6 (48.3 – 75.9)	50.66
	≥ 3	39.1 (19.76 – 77.2)	23.4 (8.73 – 63)	23.4 (8.73 – 63)	9.95

Diameter of metastasis (cm)	≤2.7	79.1 (68.3 – 91.5)	67.7 (54.6 – 84)	67.7 (54.6 – 84)	50.66
	>2.7	45.8 (29.9 – 70.3)	26 (12.8 – 53)	26 (12.8 – 53)	10.91

BED – biological effective dose

Table S4. Overall survival depending on risk factors

**Predictor**		**1 year**	**2 year**	**3 year**	**Median**
Number of metastases, *n*:	1	92.5 (84.6 – 100)	74.6 (61.4 – 90.5)	60.5 (45.5 – 80.4)	36.66
	≥2	95.4 (89.4 – 100)	61.7 (48.1 – 79.2)	31.3 (19 – 51.8)	25.69
	wild type	94.5 (88.6 – 100)	75.1 (63.8 – 88.5)	54.2 (40.8 – 72)	38.14
RAS mutation,	mutant	92.6 (83.2 – 100)	54.8 (38.1 – 78.8)	27.8 (14.3 – 54.1)	24.08
	metachronous	100 (100 – 100)	84.6 (71.8 – 99.7)	63.2 (46.5 – 85.8)	43.17
Type of metastases	synchronous	90.3 (82.5 – 98.8)	58.3 (45.8 – 74.3)	35.3 (23.3 – 53.5)	25.69
	≤2.6	96.1 (91 – 100)	83 (72.9 – 94.5)	51.6 (38 – 70.1)	36.66
Diameter of metastasis (cm)	>2.6	90.6 (81 – 100)	41.7 (26.6 – 65.2)	33.7 (19.7 – 57.6)	22.54

BED – biological effective dose
